# Control of human papillomavirus gene expression by alternative splicing

**DOI:** 10.1016/j.virusres.2016.11.016

**Published:** 2017-03-02

**Authors:** Sheila V. Graham, Arwa Ali A. Faizo

**Affiliations:** MRC-University of Glasgow Centre for Virus Research; Institute of Infection, Immunity and Inflammation; College of Medical Veterinary and Life Sciences, University of Glasgow, Garscube Estate, Glasgow G61 1QH, Scotland, UK

**Keywords:** SA, splice acceptor site, SD’, splice donor site, ESE, exonic sequence enhancer, ESS, exonic sequence silencer, ISE, intronic sequence enhancer, ISS, intronic sequence silencer, PTB, polypyrimidine tract binding protein, CPSF, cleavage and polyadenylation specificity factor, CstF, cleavage stimulatory factor, snRNP, small nuclear ribonucleoprotein particle, hnRNP, heterogenous nuclear ribonucleoprotein, Human papillomavirus, Life cycle, Alternative splicing, E2, SR protein, hnRNP

## Abstract

Human papillomaviruses possess circular double stranded DNA genomes of around 8 kb in size from which multiple mRNAs are synthesized during an infectious life cycle. Although at least three viral promoters are used to initiate transcription, viral mRNAs are largely the product of processing of pre-mRNAs by alternative splicing and polyadenylation. The HPV life cycle and viral gene expression are tightly linked to differentiation of the epithelium the virus infects: there is an orchestrated production of viral mRNAs and proteins. In this review we describe viral mRNA expression and the roles of the SR and hnRNP proteins that respectively positively and negatively regulate splicing. We discuss HPV regulation of splicing factors and detail the evidence that the papillomavirus E2 protein has splicing-related activities. We highlight the possibility that HPV-mediated control of splicing in differentiating epithelial cells may be necessary to accomplish the viral replication cycle.

## Highlights

• Alternative splicing is a key cellular mechanism controlling HPV gene expression.
• Many cellular SR proteins and hnRNPs have been identified that bind and control production of viral mRNAs.
• HPV16 E2 protein controls expression of SR proteins and has splicing-related functions.
• HPV16 infection through its regulatory effects on splicing factors may significantly alter cellular gene expression and cellular metabolism.

## 1 Introduction

Papillomaviruses comprise an ancient and ubiquitous virus family that infects humans and other animals (Bravo and Félez-Sánchez, 2015). Human papillomaviruses comprise the largest group of papillomaviruses. There are over 200 different HPV genotypes identified to date based on full genome sequencing (2012). The majority of HPV subtypes are classified under the alpha- and beta-HPV groups while a few other HPV subtypes have been classified under the gamma, mu and nu genera (Bernard et al., 2010, de Villiers et al., 2004). In general, alpha HPVs infect mucosal epithelia while beta HPVs infect external cutaneous epithelia. HPV infection causes a range of benign conditions such as condyloma acuminata (genital warts), focal epithelial hyperplasia, common warts, plantar warts and pigmented warts (Cubie, 2013, Doorbar et al., 2015). Infection with HPV is usually transient and the majority of infections are cleared by the immune system (Stanley, 2012). However, in the case of some HPVs, if infection becomes persistent this may lead to tumour progression (Bodily and Laimins, 2011). Around forty alpha HPVs infect the anogenital epithelium. Of these, up to fifteen genotypes are so-called “high-risk” HPVs (HR-HPVs) because they are associated with a range of cancers including cervical and other anogenital cancers and oropharyngeal cancers (Cubie, 2013). HPV type 16 is the most prevalent HR-HPV responsible for 55% of cervical cancers. After chlamydia, it is the second most prevalent sexually transmitted infectious agent worldwide. In the developed world the incidence of certain anogenital and oropharyngeal cancers has increased significantly over the last decade (Gillison et al., 2015). Thus, the medical importance of HPV is clear. Vaccines against HPV16 and HPV18, the next most prevalent HR-HPV and the genital wart-causing, non-oncogenic HPVs 6 and 11, have been available for eight years. However, these are prophylactic and cannot protect the very large numbers of people worldwide who are already infected and at risk of serious disease. Understanding viral gene regulation and its relationship to the infected epithelium is a key goal to allow development of novel antiviral strategies in future.

## 2 The human papillomavirus life cycle

### 2.1 Human papillomavirus entry

Papillomaviruses have a small circular double-stranded DNA genome of around 8 kb that is packaged in an icosahedral protein shell. The current model of the capsid is that it comprises 72 pentamers of L1 protein, with L2 protein monomers inserted at the centres of the pentamers (Buck and Trus, 2012). HPVs enter basal cells of the cutaneous or mucosal epithelia through trauma or microabrasions, but particularly in the cervical epithelium initial infection may occur in the single cell layer between the ecto and endocervix (Herfs et al., 2012, Mirkovic et al., 2015) before transfer to the multi-layered epithelium. For most HPVs studied, the L1 capsid protein attaches to heparan sulphate proteoglycans on the basement membrane or the basal epithelial cell surface and virus enters into the cell by micropinocytosis (Sapp and Bienkowska-Haba, 2009). The entry receptors for HPVs are not fully understood but may involve a number of proteins including epidermal growth factor receptor (EGFR), integrins, tetraspanin-enriched membrane microdomains, laminins and the annexin-A2 heterotetramer (Raff et al., 2013). HPV travels in the cytoplasm from endosomes to the *trans*-golgi network and reaches the nucleus approximately 24 h after initial attachment of virus. Recent evidence suggests that the viral genome enters the nucleus following breakdown of the membrane during mitosis (DiGiuseppe et al., 2016). Inside the nucleus, initial amplification of the virus genome to 50–100 copies occurs through expression of E1 and E2 viral replication proteins (Ozbun, 2002). During division of infected cells, E2-binding proteins such as cellular Brd4 can tether viral episomes to cellular chromatin to allow equal segregation of viral genomes into daughter epithelial cells (Wu and Chiang, 2007). Upon basal cell division, infected daughter cells may stay in the basal layer or may become transit amplifying cells that begin to move into the suprabasal epithelial layers (Doorbar, 2005).

### 2.2 Human papillomavirus replication and epithelial differentiation

**Fig. 1:**
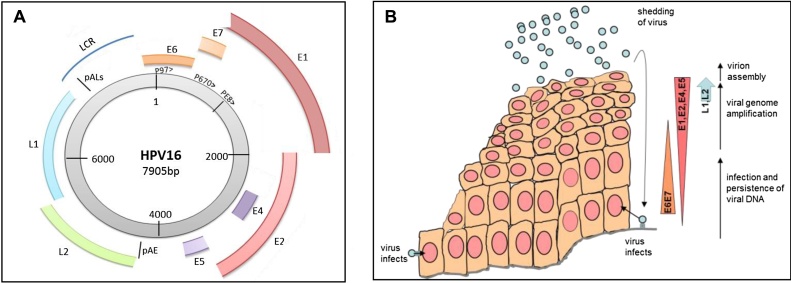
A Schematic diagram of the HPV 16 genome. The double stranded DNA genome is shown as a gray shaded hoop. Numbers indicate positions on the genome. Promoters P_97,_ P_670_ and P_E8_ are indicated with chevrons. Oncogenes E6 and E7 are indicated as orange colored arcs, replication factors E1 and E2 are in red, regulatory proteins E4 and E5 are in lilac and capsid proteins L1 and L2 are in green and blue arcs respectively. LCR (blue curved line), long noncoding region. pAE, position of the early polyadenylation site. pALs, position of the two late polyadenylation sites (Milligan et al., 2007). B. Schematic diagram of the HPV16 life cycle in a differentiating epithelium. Viruses are show as light blue circles. Keratinocytes are in light orange color. Nuclei are colored pink. The basement membrane is drawn with a gray line. The key events in the virus replication cycle are indicated to the right hand side of the diagram of the epithelium together with a schematic diagram of the gene expression program of the virus within the infected epithelium. Shading on the arrows represents the quantity of expression of each protein subset during the virus replication cycle.

The HPV replication cycle is tightly linked to host cell differentiation. The virus displays a tightly orchestrated gene expression program that results in epithelium stratum-specific production of viral proteins (Doorbar, 2005). The HPV genome can be categorized into three parts: the long control region (LCR), the early region and the late region (Fig. 1A). The LCR contains promoter sequences that direct transcription of both the early and late genes (Bodily and Laimins, 2011) and cis-acting sequences that regulate polyadenylation and viral late mRNA stability (Graham, 2008). Early mRNAs are polyadenylated at the early polyadenylation site, while late mRNAs are polyadenylated at one of two alternative polyadenylation sites in the LCR (Milligan et al., 2007). Control of read-through of the early polyadenylation site seems to constitute the major switch signal from early to late gene expression (Johansson and Schwartz, 2013). The early region contains seven open reading frames that encode the proteins E1, E2, E3, E4, E5, E6, E7, and E8, which carry out regulatory functions. Only E6 and E7, and possibly E1 and E2 (there is insufficient data to be sure of the sites of E8 expression at present) proteins are truly early proteins that can be detected in basal epithelial cells (Doorbar, 2005). E1, E2, E4, and E5 are expressed in the suprabasal layers and can be considered intermediate proteins. (Fig. 1B). In fact, maximum expression of the E1 and E2 viral replication and transcription factors is found in the mid to upper epithelial layers (Coupe et al., 2012, Xue et al., 2010). E4 protein is the first, and most abundant, late protein to be expressed in the mid to upper layers of the epithelium in the replicative stage of HPV infection (Doorbar et al., 1997, Middleton et al., 2003) and it is likely that this is also the location of maximum E5 expression (DiMaio and Petti, 2013). At least for HPV16, the late structural proteins L1 and L2 that form the virus capsid are expressed only in the final stages of cellular differentiation in the uppermost, granular layer of the epithelium where viral DNA is packaged in the capsid to be released to infect other cells (Fig. 1B) (Graham, 2010).

### 2.3 Interaction of HPVs with the epithelium

In an uninfected epithelium, the suprabasal cells do not divide, but undergo differentiation to eventually form the highly keratinized squames that comprise the epithelial barrier to the environment (Taylor et al., 2009). HPV-infected epithelia also display differentiation, but the process is somewhat abrogated by the presence of the virus. In particular, expression of the viral E6 and E7 proteins in the lower to middle epithelial layers triggers the differentiating cells of the suprabasal layers to re-enter S-phase. Although this misregulation would normally induce apoptosis, E6 protein inhibits this process by degrading p53 (Bodily and Laimins, 2011). Thus, the HPV-infected dividing cells of the mid layers of the epithelium can support viral DNA replication through recruitment of an E2/E1 complex to the viral origin of replication. This in turn recruits the cellular DNA replication machinery (McBride, 2013). Replication in the suprabasal layers generates many thousands of copies of progeny HPV genomes. E4 seems to play a role in priming the infected, differentiated epithelial cells to release newly formed virions by restructuring cytokeratin filaments (Doorbar, 2013). It may also contributed to genome amplification and enhance virion synthesis (Doorbar et al., 2015). E5 is also expressed late in infection. Its various roles during the infectious life cycle have been difficult to elucidate due to the very low levels of expression of this small protein. However, a major role of E5 is repression of MHC presentation of viral peptides to help avoid immune detection (DiMaio and Petti, 2013). Interactions with growth factor receptors EGFR (mainly HPVs) and PDGFR (mainly bovine papillomaviruses) indicates that E5 can feed into, and modify, growth control and cell cycle pathways (DiMaio and Petti, 2013). Finally, in the granular layer of the epithelium the L1 and L2 capsid proteins are produced.They encapsidate newly synthesized viral genomes to produce many thousands of progeny viruses which can initiate new infections (Fig. 1B) (Buck and Trus, 2012).

Owing to the complex interplay between the differentiating epithelium and the HPV replication cycle, pathogenicity of HPV is likely due to specific regulatory interactions between viral proteins and host cells factors. Over the last decade, it has become clear that RNA processing factors especially splicing factors are an integral part of these interactions and viral splicing control is the focus of this review.

## 3 Splicing

**Fig. 2:**
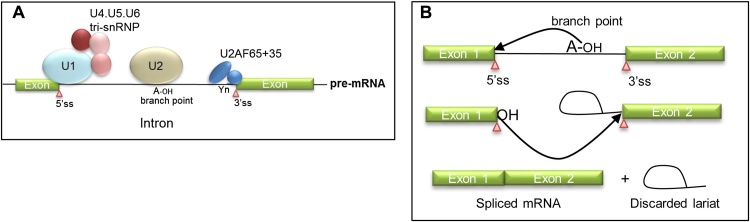
A Basic features of a pre-mRNA recognised by the early splicing complex. Exons are shown as green boxes and intron the with a black line. Pink triangles indicate 5′- and 3′- splice sites (ss). The intron branch point is indicated with A-OH. The 3′ intronic polypyrimidine tract is shown as Yn. U1 snRNP is shown as a light blue oval. U2 snRNP is shown as a light beige oval. The U4.U5.U6 tri-snRNP is shown as red/pink spheres. U2AF 65 and 35 kDa dimer is shown as a blue oval and a circle. B proteins complexes are not drawn to scale. The catalytic steps in splicing. Exons are shown as green boxes and the intron as a black line. Splice sites and branch point annotations are as above. In the first step of the splicing reaction the 2′ —OH of the branch point adenosine attacks and breaks the phosphodiester bond in the RNA backbone at the 5′ splice site and a new bond is created between the 5′ splice site and the branch point to form a lariat intermediate structure. The second step involves another exonucleolytic attack of the 5′ splice site −OH onto the 3′ splice site. The exons are spliced together and the intron lariat is discarded.

The primary transcript (pre-mRNA) of a gene that emerges from RNA polymerase II upon transcription undergoes processing to form a mature messenger RNA (mRNA). These processing events occur co-transcriptionally and include capping, polyadenylation and splicing (Moore and Proudfoot, 2009). Splicing is a basic cellular process required for expression of the majority of metazoan genes. During splicing, introns are removed from the primary transcript and the protein-coding exons are spliced together (Black, 2003). A macromolecular ribonucleoprotein complex called the spliceosome carries out these reactions through recognition of 5′ and 3′-splice sites that mark exon/intron junctions in the pre-mRNA, a “branch point” sequence, and a polypyrimidine tract within the intron upstream of the 3′-splice site (Fig. 2A) (Wahl et al., 2009). These sequences are the “landing pads” for the small nuclear ribonucleoprotein particles (snRNPs U1, U2, U4, U5 and U6) that make up the spliceosome that carries out the splicing reaction (Wahl et al., 2009). Splicing occurs through a set of well-defined steps. First U1 snRNP bind to the 5′-splice site through complementarity between the snRNA of U1 snRNP and the splice site itself, and the binding is stabilised through snRNP proteins such as U1C. Next the branch point binds splicing factor 1 (SF1/BBP1), the polypyrimidine tract binds U2AF65 while the 5′-splice site binds its heterodimer partner, U2AF35. Next, SF1 is replaced upon the branch point sequence by U2 snRNP whose binding is stabilised by U2AF and the U4.U5.U6 tri-snRNP that joins the complex. U1 and U4 snRNPs are released and U2, U5 and U6 provide the active site for splicing (Fig. 2A). The two-step enzymatic reaction involves release of the upstream exon and lariat formation of the intron back to the branch point followed by joining of the exons and release and degradation of the lariat intermediate (Fig. 2B) (Papasaikas and Valcárcel, 2016).

### 3.1 Control of splicing

**Fig. 3:**
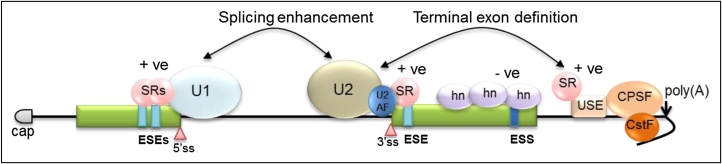
Splicing control by SR proteins and hnRNPs. SR proteins can enhance splicing by aiding the formation and stability of splicing complexes. In this case, for splicing enhancement, only interactions with U1 and U2 snRNPs are shown. However, SR proteins bound to exonic sequence enhancers (ESEs) can influence formation of the various U-snRNP complexes that form during a splicing reaction (Howard and Sanford, 2015). hnRNPs bound to exonic sequence silencers (ESSs) can counteract the activities of SR proteins (Eperon et al., 2000). A possible route of terminal exon definition is also shown where SR proteins bind to a polyadenylation upstream sequence element (USE) and create interactions from there to the upstream 3′ splice site to enhance U-snRNP recruitment (Howard and Sanford, 2015). Green boxes indicate exons. Light blue vertical boxes indicate ESEs. Dark blue vertical boxes indicate ESSs. Introns and 5′ and 3′ untranslated regions are indicated with a black line. Pink triangles indicate 5′- and 3′- splice sites (ss). U1 snRNP is shown as a light blue oval. U2 snRNP is shown as a light beige oval. U2AF dimer is shown as a blue circle. SR proteins are represented by pink spheres. hnRNPs are indicated with lilac ovals. The mRNA cap is shown as a gray bullet. The CPSF and CstF polyadenylation complexes are represented as light and dark orange spheres. A beige box indicated a polyadenylation USE in the 3′ untranslated region. A downward black arrow indicates the polyadenylation site.

The huge complexities of the splicing machinery facilitate splice site recognition, but other regulatory mechanisms are important for efficient and accurate splice site detection. Exons contain cis-acting sequences, called exonic sequence enhancers (ESEs), which influence splicing efficiency. They do this by binding splicing enhancing serine-arginine rich proteins (SR proteins). SR proteins are conserved in eukaryotes and are present mainly in the nucleus although some can shuttle to the cytoplasm (Busch and Hertel, 2012). Very early in the splicing reaction U1 and U2 snRNP early splicing complexes can assemble across introns to direct the spliceosome to the correct splice sites (Fig. 2). Early splice complex formation and stabilisation is controlled by SR proteins binding to ESEs. For example, interaction between SR proteins and U2AF35 stabilises U2 snRNP bound to the 3′ end of an intron. Cross-exon or intron splicing complexes are cooperatively stabilised by interactions between U1 snRNP and U2AF and SR proteins (Fig. 3). Moreover, SR proteins are implicated in recruitment of multiple splicing factors during the process of spliceosome assembly and are key players in the catalytic steps of the splicing reaction (Howard and Sanford, 2015). For complex genomes, SR proteins “define” exons in pre-mRNAs to establish exon-intron boundaries. SR proteins bound to ESEs connect the 3′ splice site at the 5′ end of an exon with the 5′ splice site at the other end of the intron and mark the sequence as an exon for retention in the mRNA (Fig. 3). They also facilitate definition and splicing of terminal exons (Howard and Sanford, 2015, Long and Caceres, 2009). They accomplish this by binding directly, or interacting with other RNA processing factor complexes tethered in the mRNA 5′ untranslated region or the 3′ polyadenylation region, to provide a “feedback” interaction with the first 5′-splice site or terminal 3′-splice site respectively in the pre-mRNA (Fig. 3) (Berget, 1995, Proudfoot, 2000).

In addition to SR proteins, the exon/intron architecture, steric hindrance and RNA secondary structure may also play a role in directing the activities of the spliceosome (De Conti et al., 2013).

### 3.2 Alternative splicing

**Fig. 4:**
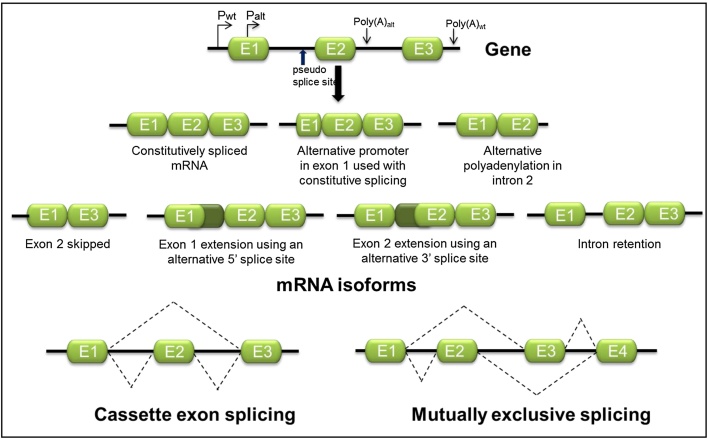
A single gene can give rise to several alternative mRNAs. At the top is shown the structure of a hypothetical three-exon, two-intron gene. Exons are illustrated in green and introns and 5′ and 3′ untranslated regions as black lines. Two alternative promoters P_wt_ and P_alt_ are shown as forward facing black arrows. Two alternative polyadenylation sites (Poly(A)_wt_ and Poly(A)_alt_) are shown as downward facing black arrows. An alternative (pseudo) 3′ splice site is indicated with a blue upward arrow. Some alternative mRNA structures that can arise from the hypothetical gene are shown. The three isoforms in the top row have undergone complete constitutive splicing. The three isoforms in the middle row are the products of alternative splicing of the gene. The bottom row shows the possible splicing patterns of a three exon gene where the middle so-called “cassette” exon can be retained or spliced out and of a four exon gene where mutually exclusive splicing can take place. In this case either exon 2 or exon 3 is included in the final mRNA isoforms produced.

In constitutive splicing all introns are removed from the pre-mRNA and every exon is present in the mature mRNA. Accurate and specific recognition of correct 5′- and 3′-splice sites is essential to ensure production of the appropriate set of mRNAs in a cell (Black, 2003). However, although consensus sequences have been determined, 5′- and 3′-splice sites are frequently found to be degenerate. This ambiguity in splice site recognition gives rise to the possibility of multiple choices of splice sites within complex pre-mRNAs (Barash et al., 2010) (Roca et al., 2013). Indeed, we now know that most mammalian pre-mRNAs can undergo regulated selection of alternative 5′ and 3′-splice site. Alternative splicing results in differential intron and exon retention, or skipping, or choice of alternative (pseudo) splice sites to alter exon size, and this process can generate several different mRNA and protein isoforms from each protein coding gene (Fig. 4) (Black, 2003, Irimia and Blencowe, 2012, Ward and Cooper, 2010). The majority of alternative splicing events comprise “cassette” exon removal from a pre-mRNA but mutually exclusive splicing is also common (Fig. 4). Probably because the HPV genome is polycistronic, several types of alternative splicing are used to generate HPV mRNAs. Cassette exon removal is seen in the case of mRNAs that skip the E4 open reading frame, e.g. E1^L1 mRNAs. Read-through versus splicing also occurs, e.g. E1^E4,E5,L2,L1 versus E1^E4^L1 mRNAs. Alternative choice of 3′ splice acceptor sites is seen in the case of the E6E7 RNAs. However, there is no evidence as yet for true mutually exclusive splicing. Although other cellular strategies exist by which different mRNA isoforms can be expressed from a single gene, including alternative promoter usage and alternative polyadenylation (Fig. 4), alternative splicing makes the greatest contribution to maximising protein production from the genomes of higher eukaryotes and viruses (Hernandez-Lopez and Graham, 2012, Ward and Cooper, 2010). Collective studies have shown that most human pre-mRNAs normally undergo extensive alternative splicing. Over 90% of human RNAs are alternatively spliced and give rise to a cellular mRNA population that can encode around 4–5 fold more proteins than there are protein-coding genes in the genome (Hallegger et al., 2010). Alternative splicing is essential for development and differentiation and organ function (Irimia and Blencowe, 2012). Mis-splicing however, is possible and it can give rise to serious health problems including cancers and genetic diseases (Scotti and Swanson, 2016).

**Fig. 5:**
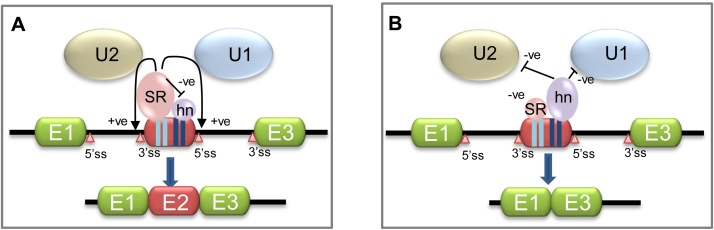
SR proteins direct alternative splicing. The red central exon in this hypothetical gene is a “cassette” exon that can be omitted from one of the mRNA isoforms generated from the gene by a failure of the spliceosome to recognise the exon boundaries efficiently. A. The mRNA isoform product of splicing is the constitutive isoform because the two introns are spliced out and the three exons are spliced together. SR proteins binding to exonic sequence enhancers (ESEs: light blue vertical boxes) act in a dominant positive manner to recruit U-snRNPs and/or increase efficiency of U-snRNP recognition of 3′ and 5′ splice sites and/or to antagonise the repressive activity of hnRNPs bound to exonic sequence silencers (ESS: dark blue vertical boxes). The number and range of SR proteins binding (usually) multiple ESEs on the exon can modulate the level of the positive effect. B. The mRNA isoform product of splicing is the alternative isoform because the central exon has not been recognised efficiently for splicing into the mRNA and only the two flanking exons are spliced together. In this case hnRNPs bound to exonic sequence silencers may exert repressive activity on U-snRNP recruitment and SR proteins enhancing activities. Green or red boxes indicate exons. Introns and 5′ and 3′ untranslated regions are indicated with a black line. Pink triangles indicate 5′- and 3′- splice sites (ss). U1 snRNP is shown as a light blue oval. U2 snRNP is shown as a light beige oval. SR proteins are represented by pink spheres. hnRNPs are indicated with lilac ovals.

Control of alternative splicing is exerted by the strength of 5′ and 3′-splice sites, the order in which exons emerge from RNA polymerase II during transcription, the pattern of RNA processing factors binding the pre-mRNA, the rate at which the gene is transcribed, and cell signaling. As mentioned above SR proteins can act positively to control constitutive splicing, and this is also true for alternative splicing (Fig. 5). Splicing can be controlled negatively by a large family of heterogeneous ribonucleoproteins (hnRNPs). hnRNPs can block exon/intron definition by interfering with assembly of the exon definition complex (Hertel, 2008). Therefore, the SR and hnRNP protein families can act antagonistically in controlling splicing (Eperon et al., 2000). Apart from ESEs, other splicing regulatory cis-active signals exist: intronic sequence enhancers (ISEs) and exonic and intronic sequence silencers (ESSs, ISSs). SR proteins generally bind the enhancer sequences while hnRNPs bind the silencers. This sequence-specific control of splicing has been termed the “splicing code” (De Conti et al., 2013).

### 3.3 SR proteins

There are nine classical SR proteins (SRSF1-9) in addition to other non-classical proteins such as SRp38 and Tra2*β* (SRSF10) (Manley and Krainer, 2010). Each SR protein is composed of at least one copy of each of two domains: 1) an RNA recognition motif and 2) a serine/arginine-rich domain (RS binding domain) (Long and Caceres, 2009). As discussed above, in constitutive and alternative splicing SR proteins control recruitment of components of the basic splicing machinery at exon-intron boundaries and mediate exon/intron definition. SR proteins bound to ESEs can increase the efficiency by which U-snRNPs detect splice sites, and this is particularly important if these sites are poorly conserved and liable to be skipped by the splicing machinery (Fig. 5) Although SR proteins usually act to enhance splicing, they have also been shown to inhibit splicing. For example, SRSF9 can inhibit recognition of a 3′ splice site (leading to exon skipping) of exon 7 B in the hnRNP A1 pre-mRNA through binding an ISS element (Simard and Chabot, 2002).

The functions of SR proteins are controlled by phosphorylation of their RS domains. Several kinases are known to phosphorylate SR proteins including Chk1, Topoisomerase (TOPO) 1 and Serine/Arginine-protein kinases (SRPK) 1 and 2 (Giannakouros et al., 2011). Phosphorylation is essential for SR protein functions in constitutive and alternative splicing, but both hypo and hyper-phosphorylated SR proteins can inhibit splicing (Zhou and Fu, 2013) meaning that site-specific or temporal alterations in phosphorylation must be a major point of control. The exact physiological roles of phosphorylated forms of SR proteins are still to be addressed. However, the suggested importance of SR protein phosphorylation includes intracellular localization and trafficking, protein–protein interactions and control of alternative splicing of mRNAs (Giannakouros et al., 2011, Long and Caceres, 2009).

SR proteins play a range of other roles in regulating gene expression including regulation of transcription elongation, mRNA nuclear export, stability and translation (Howard and Sanford, 2015). Indeed, it seems likely that SR proteins may have much broader relevance to normal cellular metabolism than simply their role in splicing regulation. As documented for the paradigm SR protein SRSF1, other functions of SR proteins include chromatin remodelling, genome stability maintenance, nucleolar stress response, cell cycle progression and apoptosis control (Das and Krainer, 2014). Current research has described some of the SR proteins as oncogenic as they have been found to be overexpressed in a range of cancers (Das and Krainer, 2014). Moreover several have been shown to possess oncogenic activity including SRSF1 (ASF/SF2), SRSF2 (SC35), SRSF3 (SRp20) and SRSF9 (SRp30c) (Fu et al., 2013, Jia et al., 2010, Karni et al., 2007, McFarlane et al., 2015). Oncogenic activity of SR proteins is due largely to their deregulation of alternative splicing of RNAs whose protein products are involved in key cellular pathways. In summary, increased SRSF levels can result in production of alternatively spliced RNA isoforms that encode key anti-apoptotic, cell proliferation and epithelial-mesenchymal transition (EMT)-inducing proteins (Das and Krainer, 2014).

### 3.4 hnRNP proteins

The hnRNP family is larger and more complex than the SR protein family. In humans, there are thirteen hnRNP protein families each of which contain several subtypes (Busch and Hertel, 2012). Exact details of how hnRNPs control splicing are understood in only a few cases. They can bind cooperatively, multimerize, and spread along exons to repress assembly of the spliceosome across adjacent introns (Fig. 3) (Busch and Hertel, 2012). In alternative splicing, they may block snRNP binding to adjacent splice sites. Importantly, SR proteins can antagonise the negative effects of hnRNP proteins on splicing perhaps by steric hindrance of hnRNP/RNA protein interactions (Fig. 5) (Eperon et al., 2000).

## 4 Splicing of HPV RNAs

**Fig. 6:**
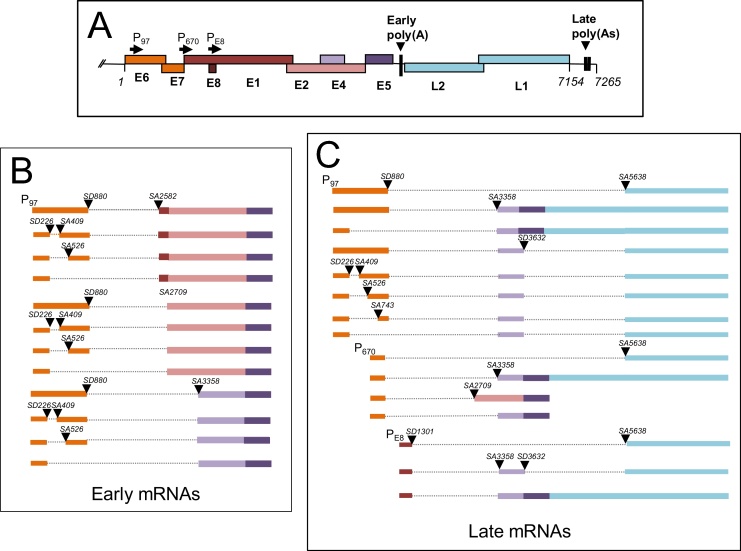
A. Diagram of the linearised HPV16 genome showing the nine open reading frames (colored boxes) the three characterised promoters (forward facing arrows) and the early and late polyadenylation sites (thick black vertical lines). B. Schematic diagram of the known HPV16 early mRNA splice sites (adapted from (Zheng and Baker, 2006)). The diagram does not indicate the 3′ ends of the RNAs listed and is not to scale. Orange colored boxes indicate E6E7 coding regions. Red/pink colored boxes indicate E1E2 coding regions. Lilac/purple colored boxes indicate E4E5 coding regions. C. Schematic diagram of the known HPV16 late mRNA splice sites (adapted from (Milligan et al., 2007) and (Chen et al., 2014)). The late polyadenylation sites are not indicated. The color scheme is the same as for B. with the addition of L1 and L2 coding regions indicated in blue. SA, splice acceptor. SD, splice donor. Arrowheads indicate splice sites. Gray dotted lines, intron sequences.

DNA viruses such as human papillomavirus (HPV) require constitutive and alternative splicing to generate mRNAs encoding the many essential proteins that are required to initiate, maintain and complete their life cycles. During HPV infection of the epithelium at least twenty different mRNAs are expressed, some of which are the products of alternative splicing (Baker and Calef, 1997, Chen et al., 2014, Chow et al., 1987a, Chow et al., 1987b, Doorbar et al., 1990, Isok-Paas et al., 2015, Ozbun and Meyers, 1997, Ozbun and Meyers, 1998, Palermo-Dilts et al., 1990, Stoler et al., 1992, Stoler et al., 1989, Tan et al., 2012, Toots et al., 2014, Wang et al., 2011). Transcript maps may be viewed at (https://pave.niaid.nih.gov/#explore/transcript_maps). SR and hnRNP proteins control viral RNA processing during infection (Graham, 2010, Johansson and Schwartz, 2013). A map of known HPV16 mRNAs is shown in Fig. 6. In addition to this infection-related control, SR and hnRNP proteins are overexpressed in HPV-associated cervical pre-cancers and cancers (Mole et al., 2009a, Fay et al., 2009) and therefore have the potential to impact HPV gene expression in tumorigenesis.

### 4.1 HPV gene expression and splicing

Most information on regulation of viral gene expression has been gathered from studies on HPV16, or the most closely related HPV, HPV31. Early in the HPV16 infectious life cycle, transcription initiates from the viral early promoter located at P_97_, and polycistronic mRNAs encoding E6 and E7 E1, E2, E8 E4 and E5 are synthesized. (Fig. 6). There is extensive splicing in the E6E7 region of the pre-mRNAs with at least four splice isoforms possible that have been confirmed in patient tissues (Chen et al., 2014, Schmitt et al., 2011). E6 full length (E6fl) is an unspliced transcript that includes the E6 and E7 open reading frames. E6*I, E6*II and E6*X (also termed E6^E7 or E6*III) are mRNAs alternatively spliced from one 5′-splice site to one of three alternative 3′-splice sites in the primary transcript (Fig. 6). Two rare splice isoforms with alternate 5′-splice sites have also been detected in HEK293 cells transfected with an E6E7 expression construct (Ajiro et al., 2012). There are four splice isoforms produced from the E1E2 region of the genome, E1^E2, E1^E2C, E8^E1, E8^E2C (Chen et al., 2014, Coupe et al., 2012, Milligan et al., 2007, Schmitt et al., 2011). These use a splice donor at either genome position 880 or 1302 and one of two splice acceptors at 2582, and 2709 (Fig. 6).

At late times of infection the viral late promoter (P_670_) is activated (Bodily and Laimins, 2011) together with a promoter located at the 5′ end of the E1 open reading frame termed the E8 promoter (Straub et al., 2015) (Fig. 6). Despite the fact that the E8^E2C protein is an inhibitor of E2 in transcription and replication, this results in increased expression of the HPV replication/transcription factors E1 and E2 that initiate vegetative viral genome amplification. The viral capsid proteins are expressed from two classes of polycistronic transcripts transcribed from the late promoter. The first one includes two splice events to give E1^E4^L1 mRNAs that are considered to encode E4 and L1 proteins and the second one includes only the E1^E4 splice event and read-through from the early region to yield an E4, E5, L2, L1 polycistronic mRNA. The packed nature of the HPV genome in terms of signal sequences that regulate transcription, splicing and polyadenylation suggests that control of mRNA production is complex. Moreover, it is clear that alternative splicing plays a major role in generating the range of mRNAs required to encode viral proteins, and that viral splicing may be regulated in a differentiation-stage specific manner.

### 4.2 SR proteins controlling HPV gene expression

The extent and complexity of alternative splicing required to produce HPV mRNAs suggests that SR and hnRNP proteins could be key regulators of the HPV replication cycle. A large body of evidence has been amassed detailing the SR and hnRNP proteins that contribute to HPV mRNA alternative splicing and we are beginning to understand some of the controlling mechanisms.

#### 4.2.1 E6 and E7 splicing

In the case of E6 and E7 RNA isoforms, the roles of SRSF1, SRSF2 and SRSF3 have been investigated. In two studies, SRSF1 was not found to control E6E7 RNA splicing while depletion of SRSF3 resulted in some reduction in E6E7 mRNA expression (Jia et al., 2009, McFarlane et al., 2015). However, a very significant reduction in E6E7 RNA expression was observed in the absence of SRSF2 (McFarlane et al., 2015). Although this splicing factor is a major positive regulator of viral oncoprotein expression, the evidence suggests that SRSF2 regulates E6E7 RNA stability rather than splicing. Nonsense mediated decay is a mechanism whereby RNAs such as the short E6E7 mRNAs, containing 3′-splice sites close to stop codons are recognised as aberrant and subject to degradation (Popp and Maquat, 2014). SRSF2 may be involved in E6E7 splicing and protect the mRNA isoforms against decay. All of the E6E7 mRNA isoforms were similarly affected in the above experiments. Of the main E6E7 RNA isoforms, E6*I appears to be the most abundant in tumour cells lines and in patient tissues (Schmitt et al., 2011, Schmitt and Pawlita, 2011). E6*I mRNA may encode an additional viral protein expressed by HR-HPVs (Yuan et al., 2012) and in depth studies have demonstrated that the putative E6*I protein appears to have antagonistic properties to E6 itself. For example, E6*I can promote apoptosis by counteracting E6fl-mediated degradation of p53 (Yuan et al., 2012). On the other hand, data from *in vitro* studies has indicated that E6*1 mRNA may allow translation of E7 by reinitiation on a downstream AUG (Stacey et al., 2000, Tang et al., 2006). In HPV16 infected keratinocytes, the balance of E6 fl (intron-containing) versus E6*I (intron removed) RNA isoform expression was demonstrated to be regulated by EGF (Rosenberger et al., 2010). EGF signaling resulted in intron inclusion (SD226–SA409) to give predominantly E6 fl mRNAs while EGF depletion shifted the balance towards intron splicing and E6*I production. In agreement with the studies mentioned above (Jia et al., 2010, McFarlane et al., 2015), SRSF1 did not play a role in regulating E6 alternative splicing. However, two transcription factors that can also control splicing, Brm and Sam68 were implicated. Brm is a component of the SWI/SNF chromatin remodeler and is proposed to regulate splicing by controlling the rate of RNA polymerase II elongation while Sam68 is a member of the STAR protein family that controls splicing through signal transduction. Interestingly, it was proposed that EGF levels in tumour cells might allow a switch to production of the E6*I mRNA isoform from which E7 protein would be more efficiently translated via reinitiation at the E7 AUG. Increased E7 expression would ensure enhanced cell cycle progression, a hallmark of HPV-associated tumour progression (Roman and Munger, 2013). Conversely, in infected normal keratinocytes EGF signaling in the basal epithelial cells would favour E6 full length production and inhibition of apoptosis of the infected cell (Rosenberger et al., 2010). Finally, another study has shown that E6 isoform production from HPV18 is controlled by another transcription factor that can also have roles in transcription-linked splicing, CCCTC-binding factor (CTCF). CTCF binds to a motif in the E2 region of the HPV18 genome and initiates a pause in RNA polymerase II transcription that favours correct splicing of the E6E7 RNA. Deletion of the CTCF binding site within the viral genome led to a significant increase in levels of the E6 and E7 oncoproteins (Paris et al., 2015). It will be interesting in future to discover if there is any link between CTCF activity and EGF signaling.

#### 4.2.2 Early RNA splicing

Most viral early RNAs are spliced from a 5′-splice site at nucleotide 880 in the E1 gene region to a 3′-splice site at nucleotide 3358 to retain the E4 open reading frame (Fig. 6). The E4 gene does not contain a start codon but this is provided through splicing of the region encoding the first five amino acids of E1 onto E4 (Roberts, 2006). The 3′-splice site located at the 5′ end of the E4 open reading frame is suboptimal due to lack of a good upstream polypyrimidine tract meaning that it should be used at low efficiency (Kajitani and Schwartz, 2015). Despite this, the spliced transcript E1^E4 that contains the E4 open reading frame is the most abundant HPV mRNA expressed during an infection (Chen et al., 2014, Schmitt et al., 2011). Analysis of SR protein binding to the HPV16 E4exon has shown that the 3′-splice site at nucleotide position 3358 (SA3358) is controlled by a complex ESE containing an *in silico*-predicted ten clusters of SRSF1 binding motifs (Somberg and Schwartz, 2010). In all, fifteen SRSF1 binding sites were identified, and mutation of these resulted in a redirection of splicing from SA3358 to a downstream 3′-splice site at nucleotide position 5639 at the 5′ end of the L1 open reading frame (Fig. 6). A subsequent study revealed that the majority of the ESE activity was due to a single SRSF1 binding site (Li et al., 2013a). Similar motifs are predicted in very similar regions of E4 open reading frames of low and high risk, mucosal and cutaneous HPVs suggesting a ubiquitous SRSF1-mediated mechanism for controlling E4 splicing. In the absence of SRSF1 enhancement of the 3358 3′-splice site, there was competition from the downstream SA5639 that is used to produce L1-encoding mRNAs (Somberg and Schwartz, 2010). These data suggest that SRSF1 controls use of SA3358 at the 5′ end of the E4 open reading frame and inhibits late mRNA production. SRSF1 also had a low level repressive effect on the splice site at the 3′ end of E4 open reading frame (SA3632) that would also result in inhibition of late mRNA splicing (Somberg and Schwartz, 2010). These observations demonstrate the positive and negative effects that a single SR protein can exert on mRNA splicing. SRSF3 also binds an ESE within the E4 open reading frame and enhances splicing at SA3358. Moreover, SRSF3 could also inhibit viral late mRNA expression but this time by stimulating polyadenylation at the early polyadenylation site (Jia et al., 2009).

Splicing of the various RNAs arising from transcription of the E1 and E2 genes has been reported but it is as yet unclear how these splicing events are controlled. However, because the RNAs use a 5′-splice site at genome position 880 or 1302 with one of two splice acceptors at 2582 and 2709, selection of one site over another must be a controlled event (Straub et al., 2015). Compared to E6E7 and E4-containing RNAs these seem to be rare RNA species, which may hamper their analysis.

#### 4.2.3 Late RNA splicing

Analyses of the early splice isoforms and their regulation were mostly carried out in tumour cells such as HeLa cells that mimic undifferentiated epithelial cells or in undifferentiated keratinocytes. These cell systems only support HPV early gene expression because keratinocyte differentiation is required for viral late protein expression. To begin to examine how SR proteins contribute to late mRNA production through alternative splicing our laboratory used siRNAs to deplete SR proteins in HPV16-infected, differentiated keratinocytes to discover which were responsible for controlling capsid mRNA and protein expression. HPV capsid protein expression is readily detected in keratinocytes that maintain wild type episomal HPV genomes (Klymenko et al., 2016). Among SRSFs 1–3, 5, and 7, depletion only of SRSFs 1 and 3 caused a change in L1 capsid protein expression. SRSF1 knock down resulted in only a small reduction in L1 expression but SRSF3 knock down caused a greater than 50% reduction in L1 levels in the cells. Conversely, SRSF3 overexpression in an undifferentiated keratinocyte population resulted in induction of L1 protein expression (Klymenko et al., 2016). Analysis of the major spliced RNAs encoding the capsid proteins revealed that SRSF3 was required for production of the spliced late E4^L1 mRNA that encodes the L1 major capsid protein because a reduction in levels of SRSF3 caused a decrease in E4^L1 mRNA levels with a corresponding increase in the unspliced L2L1 mRNA that encodes the L2 minor capsid protein (Klymenko et al., 2016). In agreement with a previous study (Somberg and Schwartz, 2010), SRSF1 also contributed to maintaining levels of the E4^L1 spliced mRNA, but had a much more significant and inhibitory effect on L2L1 RNA levels. The data implicate SRSF3 as a key direct regulator of viral late gene expression in differentiating keratinocytes.

SRSF9 (SRp30c) has also been implicated in enhanced splicing of HPV16 late transcripts (Somberg et al., 2011). In undifferentiated HeLa cells, SRSF9 inhibited splicing at SA3358 at the 5′ end of the E4 open reading frame resulting in redirection of splicing downstream to SA5639 at the 5′ end of the L1 open reading frame. SRSF9 was also shown to overcome suppression of SA5639 via neutralisation of splicing silencers in the L1 open reading frame (Somberg et al., 2011) resulting in L1 RNA production. Finally, overexpression of SRSF9 induced levels of a rare mRNA called L1i (E1^L1) (Somberg et al., 2011), which can be detected in differentiated keratinocytes (Milligan et al., 2007) by promoting skipping of the E4 exon (Somberg et al., 2011). The L1 coding region contains an ESE whose positive effect on splicing to SA5639 can be overridden by hnRNP A1. While the proteins that bind are not yet elucidated they could be an essential regulator of L1 mRNA splicing (Zhao et al., 2007a).

### 4.3 hnRNP proteins controlling HPV gene expression

The hnRNP protein family has also been shown to control HPV16 mRNA splicing. Both early and late mRNAs are under hnRNP control. Expression of the viral oncoproteins is controlled by hnRNP A1 which activates splicing between the first pair of splice sites (SD226 and SA409) in viral E6E7 pre-mRNAs. As described above E6E7 mRNA isoform expression is regulated by EGF-controlled alternative splicing (Rosenberger et al., 2010). EGF depletion is linked to the activities of hnRNPs A1 and A2 and favours intron splicing leading to expression of the E6*I isoform (Rosenberger et al., 2010). Evidence also points to a role for hnRNP A1 in viral late mRNA expression. hnRNP A1 can bind AG-rich splicing silencer elements in the HPV16 L1 coding region (Table 1) and counteract the activity of SR proteins bound at L1 ESEs to suppress the use of the HPV16 late 3′-splice site SA5639 (Zhao et al., 2007a, Zhao et al., 2004). hnRNP C1 appears to bind the viral early 3′ untranslated region and activate use of the 5′-splice site SD3632 at the 3′ end of the E4 open reading frame resulting in late mRNA production (Dhanjal et al., 2015). Conversely, hnRNP D has been shown to bind to two AUAGUA motifs in an ESS element adjacent to SD3632 that controls late mRNA splicing (Li et al., 2013b). Indeed, it has been proposed that hnRNP C1, together with hnRNP D and hnRNP A2/B1, form a complex on this splicing silencer, but hnRNP C1 activity is dominant and counteracts the hnRNP D and hnRNP A2/B1-mediated ESS-induced suppression of SD3632 leading to late mRNA splicing (Dhanjal et al., 2015, Li et al., 2013b). Polypyrimidine tract binding protein (PTB, hnRNP I) has also been reported to activate splicing from SD3632, perhaps by competing with the other hnRNP proteins that suppress use of this splice site (Somberg et al., 2008). hnRNP H has been implicated in stimulating HPV16 early polyadenylation through a G-rich enhancer element in the L2 coding region (Öberg et al., 2005) and limiting viral late gene expression. hnRNP H could also antagonise viral late mRNA splicing, especially if it promoted cooperative binding of hnRNP proteins on the L2L1 exons, but this possibility remains to be investigated. Finally, hnRNPs E1, E2 and hnRNP K were found to bind HPV16 L2 mRNAs. Although splicing was not affected by these hnRNP proteins, they inhibited late mRNA translation in *in vitro* studies (Collier et al., 1998).

### 4.4 Terminal exon definition

So far, no data exist on how the 5′-most exon in any HPV transcript is defined. It is entirely possible that some of the proteins already discovered to bind viral RNAs could play this sort of role in splicing regulation. For example, for HPV16, proteins bound to the cap, or 5′ untranslated region, of viral mRNAs could form a cross-exon complex with the first E6 exon/intron junction (SD226) in transcripts synthesized from P_97_, or the E4 exon in transcripts initiated from P_670_. More information is available regarding possible mechanisms of 3′ terminal exon definition, both for the early mRNAs that terminate at the early polyadenylation, and those late mRNAs that terminate at the late polyadenylation sites of HPV16. The early polyadenylation site is inherently weak because the cis-acting sequences that bind the CPSF and CstF polyadenylation complexes are of poor consensus. However, polyadenylation complex formation is strengthened by RNA binding proteins that form a complex on a 57 nucleotide U-rich region in the early 3′ untranslated region. Proteins that bind this region include hnRNP C1/C2, PTB (hnRNP I) and the polyadenylation factors CPEB1 and hFip1 (Zhao et al., 2005). Recently, HPV E2 has also been shown to bind the CPSF-CstF polyadenylation complex to reduce efficiency of HPV early polyadenylation leading to transcription read-through to the late region and production of viral late mRNAs (Johannson et al., 2012). Since E2 can bind SR proteins including SRSF1 (Jang et al., 2015, Muller et al., 2012), it is possible that E2 can define the early terminal exon, E4, through an E2-containing polyadenylation complex even if the complex has the potential of inhibitory activity for early polyadenylation. Moreover, E4 ESE sequences that bind SRSF1 are known to influence efficient use of the early polyadenylation site (Rush et al., 2005, Somberg and Schwartz, 2010) thus highlighting the connection between terminal exon-bound proteins and polyadenylation.

At the end of the L1 open reading frame, and spanning the start of the late 3′ untranslated region, is a 79 nt RNA element termed the negative regulatory element (NRE) or late regulatory element (LRE) (Graham, 2008). The element is a conserved feature of papillomaviruses (Zhao et al., 2007b), and inhibits late gene expression in undifferentiated epithelial cells. For HPV16, it has been proposed that the LRE may enhance late polyadenylation, but another mechanism may involve formation of an exon definition complex composed of U1 snRNP, U2AF and SRSF1 on the element. Such a complex could mimic a mini-intron and negatively regulate L1 exon definition (Furth et al., 1994, McPhillips et al., 2004). Interestingly, the element also binds hnRNP A1 which might be expected to counteract the activity of the SRSF1 complex (Chuen-Im et al., 2008). The effect of the balance between splicing stimulatory and inhibitory factors in HPV mRNA terminal exon definition requires further investigation.

## 5 HPV regulation of SR protein activity

Demonstrating their key roles in HPV infection, SR proteins appear to be upregulated during the HR-HPV life cycle in an epithelial differentiation-specific manner. For example, SRSF1, 2 and 3 levels are significantly increased in the mid to upper layers of infected keratinocytes and in tissue samples from patients with low grade cervical lesions that represent transient HPV infection (Mole et al., 2009a). This is controlled by the HPV E2 transcription factor (Mole et al., 2009a), which binds and trans-activates the promoters of the SR protein genes (Klymenko et al., 2016, Mole et al., 2009b). The observed high levels of SR proteins in the nuclei of cells of the mid to upper layers of the infected epithelium correlate with peak levels of E2 that are also detected in these cells (Coupe et al., 2012, Klymenko et al., 2016, Xue et al., 2010). It could be argued that E2 activation of SR proteins in the mid to upper epithelial layers would be detrimental to viral replication. High levels of SRSF1 should activate alternative splicing from SD880 to SA3358 at the 5′ end of the E4 open reading frame, thus precluding expression of mRNAs encoding E2. It is possible that other SR proteins or hnRNPs that bind the E2 region of viral pre-mRNAs compete with E4 splice site selection to allow expression of E2, but E2 splicing regulatory factors have not yet been reported. It is worth noting that very low levels of E2 mRNAs compared to E4 mRNAs are detected in HPV-infected patient tissues (Schmitt et al., 2010, Chen et al., 2014). In alternative splicing the rule seems to be that for mRNAs subject to alternative splicing, the first pair of 5′- and 3′- splice sites intron that emerge from RNA polymerase II are preferentially chosen for splicing over subsequent sites. Thus, in mRNAs initiating at P_670_ of HPV16 the intron between SD880 and SA2582 or SA2709 should be removed in preference to the intron between SD880 and SA3358. Leaky splicing control could result in read-through to the E4 splice site and competition between architecture (first-splice-first) and a dominant E4 ESE could yield the observed low levels of E2 mRNAs and high levels of E4-encoding mRNAs. E2 control of SR proteins could be beneficial to completion of the virus replication cycle. SR protein expression is greatest in basal epithelial layers but expression levels decrease to a low level in normal, uninfected, differentiated keratinocytes (Fay et al., 2009, Jia et al., 2010, Mole et al., 2009a). This change is expected because differentiated epithelial cells are beginning to shut down nuclear functions such as splicing. However, in infected, differentiated keratinocytes the viral late proteins such as E4 and L1 are express from spliced mRNAs. Therefore, HPV-mediated upregulation of key splicing factors, for example factors that bind the L1 ESE (Zhao et al., 2007a), could facilitate efficient and accurate splicing in the infected differentiating epithelial cell. SRSF3 seems to be a key SR protein driving late gene expression (Klymenko et al., 2016), but SRSF3 also regulates other SR proteins and has been designated a master regulator of splicing (Ajiro et al., 2016, Änkö et al., 2012). This means that HPV up-regulation of SRSF3 could have quite global effects on constitutive and alternative splicing in differentiated keratinocytes, even to the extent of inducing a de-differentiation or pre-neoplastic phenotype. An intriguing possibility emerges that HPV E2 control of SR protein expression during and infectious life cycle could contribute to HPV-associated tumour progression.

### 5.1 E2 as a splicing factor

HPV E2 protein plays a crucial role in the HPV life cycle and pathogenicity due to its involvement in viral genome replication, transcription and segregation (McBride, 2013). It consists of three functional regions, an N-terminus which is the transactivation domain, a C-terminal DNA binding domain and a hinge region that links the N- and C-termini (Hegde, 2002). The interactome of E2 proteins with cellular proteins has recently been analysed to give a clearer insight into the wide range of E2 activities (Jang et al., 2015, Muller and Demeret, 2012). E2 can interact with SR proteins SRSF1, 2, 4, 5 and 7 (Bodaghi et al., 2009, Jang et al., 2015, Lai et al., 1999, Muller and Demeret, 2012). E2 protein also interacts with key components of the spliceosome and other cellular RNA processing factors (Graham, 2016). While early studies on the low risk HPV5 E2 serine-arginine-rich hinge domain showed that it could facilitate splicing (Lai et al., 1999), a later study found that it could not and suggested instead that HPV16 E2 may have splicing repressive activity (Bodaghi et al., 2009). This study also showed that E2 can bind RNA directly via its C-terminal domain (Bodaghi et al., 2009). E2 appears to have many of the properties of a protein that can nucleate protein–protein and protein-RNA interactions. Although further studies are required to elucidate the role of E2 in splicing regulation, it is clear that E2 protein could affect splicing in the infected cell simply through its ability to recruit cellular splicing factors to RNA in a similar manner to its recruitment of polyadenylation factors (Johannson et al., 2012). Indeed, a study using exon array analysis revealed that overexpression of E2 in U2OS osteosarcoma cells resulted in significant changes in cellular alternative splicing (Gauson et al., 2014). This effect could be due to E2 transcriptional upregulation of SR protein expression. However, increased SR protein levels were not observed in the study. In fact, U2OS cells, like many cancer cell lines, already express high levels of SR proteins (Graham, unpublished data) perhaps negating the transcriptional trans-activation effect of E2. The most likely and exciting explanation is that E2 alters regulation of cellular alternative splicing.

### 5.2 SR protein phosphorylation during infection

SR protein activity is controlled through phosphorylation by serine-arginine protein kinases (SRPK) 1 and 2, Chk1 and Topoisomerase 1 (Zhou and Fu, 2013). Phosphorylation/dephosphorylation cycles are crucial in splicing and nuclear export of SR proteins. As well as SRSF proteins, SR protein kinases may also be regulated during HPV infection. For example, HPV1 E4 colocalises with and regulates SRPK1 in infected keratinocytes (Prescott et al., 2014). E4 binding to SRPK1 alters its ability to phosphorylate SR proteins *in vitro* suggesting that HPV infection can control not only SR protein levels, but also their various cellular activities (Prescott et al., 2014). Another study made use of the adenovirus E4orf4 protein to alter HPV16 mRNA production to favour production of the viral E4^L1 spliced late mRNA (Somberg et al., 2009). E4orf4 interacts with SR proteins but can also bind the phosphatase PP2A. Indeed, E4orf4 overexpression in HeLa cells resulted in loss of SR protein phosphorylation (Kanopka et al., 1998). Using HPV subgenomic expression plasmids in HeLa cells, it was found that overexpression of under-phosphorylated SR proteins induced viral splicing to the major late 3′ splice site SD5639 and production of the late E4^L1 mRNA (Somberg et al., 2009). The phosphorylation status of SR proteins during HPV infection of the epithelium remains to be studied in detail. At least for SRSF1, an increase in phosphorylation was detected upon differentiation of HPV16-infected W12 cervical epithelial cells, but we have not yet determined the downstream effects of this alteration on SRSF1 and the viral life cycle (McPhillips et al., 2004). Some drugs are available that inhibit SRPK1. These small molecule inhibitors have been shown to successfully inhibit replication of human immunodeficiency virus (HIV), hepatitis C virus (HCV) and Sindbis virus (Hernandez-Lopez and Graham, 2012). It will be informative to use these compounds to investigate any effects on HPV splicing patterns.

## 6 Conclusions

This review details many studies whose conclusions support the hypothesis that cellular splicing regulatory mechanisms, and splicing factors such as SR proteins and hnRNPs, are essential for controlling HPV gene expression. Moreover, an emerging hypothesis is that HPV infection controls cellular splicing in order to complete the viral life cycle in the differentiating epithelium. The necessary link between the HPV life cycle and epithelial differentiation must be considered important in elucidating mechanisms regulating viral mRNA production. Activity of any RNA regulatory element should be responsive to the components of the protein complex that forms upon it. The positive and negative effects of the various RNA-binding factors on the multiple papillomavirus elements that regulate viral RNA processing could be altered during the epithelial differentiation program due to changed levels of these factors between undifferentiated and fully differentiated epithelial cells. For example, in differentiated keratinocytes an HPV-induced increase in levels of key SR proteins could alter the composition or efficiency of formation of splicing complexes (or polyadenylation complexes) leading to appropriate late splicing events, stimulation of late terminal exon definition and late polyadenylation. This would lead directly to efficient viral late protein production in the appropriate (upper) epithelial layers.

Alternative splicing is essential for the life cycles of nuclear-replicating viruses because it allows expression of multiple proteins from a small genome. It is clear that alternative splicing is required for the HPV replicative life cycle because it is only through alternative splicing that mRNAs encoding the E1 and E2 viral replication factors are expressed, the correct balance of E6, E6 isoforms and E7 proteins are synthesized, and capsid protein synthesis is coordinated with epithelial differentiation. Alternative splicing is also implicated in HPV-associated cancer progression due to expression of the various E6 mRNA isoforms that encode the viral oncoproteins whose overexpression leads to tumorigenesis. Regulation of viral gene expression at the level of alternative splicing still requires further study and number of important unanswered questions remain to be addressed. For example, how does the architecture of the various viral pre-mRNAs allow alternative splicing given the possibility of steric hindrance between splicing complexes formed at intron-exon junctions on short introns such as those found in E6E7 isoform RNAs? The viral late mRNAs contain unusually long exons (L1 exon: 1.5 kb, L2L1 bicistronic exon: 2.9 kb) that likely contain pseudo-splice sites and alternative polyadenylation sites. How are these exons defined for accurate splicing? Some viral mRNAs (e.g E6 fl mRNA) are predicted to contain intronic sequences which would normally preclude their nuclear export and translation. Other viruses express proteins that ensure efficient export of viral intron-containing transcripts (Harris and Hope, 2000) but there is little information on how HPV ensures export of these mRNAs. Further, the role of the HPV E2 protein, and its potential roles in regulating viral and cellular splicing, has yet to be fully elucidated. Understanding HPV splicing could lead to development of novel therapeutic approaches to inhibit viral replication or virally-induced tumour formation in future (Graham, 2010, Hernandez-Lopez and Graham, 2012).

## Figures and Tables

**Table 1:** RNA binding proteins and their effects on their target HPV16 RNAs. If it has been identified, the target splice acceptor (SA) or splice donor (SD) site is listed.

RNA binding protein	Target RNA	Effect	Reference
SRSF1	E4 E4 LRE	SA3358 enhancement SD3632 suppression Repression	Li et al. (2013a); Somberg and Schwartz (2010) Li et al. (2013a); Somberg and Schwartz (2010) McPhillips et al. (2004)
SRSF2	E6E7	Enhancement via RNA stability	McFarlane et al. (2015)
SRSF3	E6E7 E4	Enhancement SA3358 suppression	Jia et al. (2009); McFarlane et al. (2015) Jia et al., 2009, Jia et al., 2010
SRSF9	E4	SA3358 suppression SA5639 activation	Somberg et al. (2011)
Sam68	E6E7	E6exon inclusion	Rosenberger et al. (2010)
Brm	E6E7	E6exon inclusion	Rosenberger et al. (2010)
hnRNPA1	E6E7 L1 LRE	E6exon exclusion SA5639 suppression Repression	Rosenberger et al. (2010) Zhao et al. (2007a); Zhao et al. (2004); Zhao and Schwartz (2007) Chuen-Im et al. (2008)
hnRNPA2/B1	E6E7 E4	E6exon exclusion Suppression/enhancement	Rosenberger et al. (2010) Li et al. (2013b); Orrù et al. (2012)
hnRNAPC1/C2	E4 Early 3′UTR	SD3632 enhancement Activation/repression	Dhanjal et al. (2015) Dhanjal et al. (2015)
hnRNPD	E4	SD3632 suppression	Dhanjal et al. (2015)
hnRNPH	L2	Enhances early polyadenylation	Öberg et al., 2003, Öberg et al., 2005
hnRNPI (PTB)	Early 3′UTR	Enhances early polyadenylation Relieves SD3632 suppression	Somberg et al. (2008); Zhao et al. (2005) Somberg et al. (2008)
CTCF	E2	RNA PolII-related control of early region alternative splicing	Paris et al. (2015)
